# CTX-M-producing *Escherichia coli* ST602 carrying a wide resistome in South American wild birds: Another pandemic clone of One Health concern

**DOI:** 10.1016/j.onehlt.2023.100586

**Published:** 2023-06-16

**Authors:** Gislaine Dalazen, Danny Fuentes-Castillo, Luiz G. Pedroso, Herrison Fontana, Elder Sano, Brenda Cardoso, Fernanda Esposito, Quezia Moura, Bianca S. Matinata, Luiz F. Silveira, Mashkoor Mohsin, Eliana R. Matushima, Nilton Lincopan

**Affiliations:** a Laboratory of Wildlife Comparative Pathology, Department of Pathology, School of Veterinary Medicine and Animal Sciences, University of São Paulo, São Paulo, Brazil; b One Health Brazilian Resistance Project (OneBR), São Paulo, Brazil; c Departamento de Patología y Medicina Preventiva, Facultad de Ciencias Veterinarias, Universidad de Concepción, Chillán, Chile; d Laboratory of Acarology, Department of Zoology, São Paulo State University, Rio Claro, São Paulo, Brazil; e Department of Clinical Analysis, Faculty of Pharmacy, University of São Paulo, São Paulo, Brazil; f Department of Microbiology, Institute of Biomedical Sciences, University of São Paulo, São Paulo, Brazil; g Federal Institute of Espírito Santo, Vila Velha, Brazil; h Zoology Museum of the University of São Paulo, University of São Paulo, São Paulo, Brazil; i Institute of Microbiology, University of Agriculture, Faisalabad, Pakistan

**Keywords:** Wildlife, ESBL, High-risk clone, Antimicrobial resistance, Genomic surveillance, WHO priority pathogens

## Abstract

Wild birds have emerged as novel reservoirs and potential spreaders of antibiotic-resistant priority pathogens, being proposed as sentinels of anthropogenic activities related to the use of antimicrobial compounds. The aim of this study was to investigate the occurrence and genomic features of extended-spectrum β-lactamase (ESBL)-producing bacteria in wild birds in South America. In this regard, we have identified two ESBL (CTX-M-55 and CTX-M-65)-positive *Escherichia coli* (UNB7 and GP188 strains) colonizing Creamy-bellied Thrush (*Turdus amaurochalinus*) and Variable Hawk (*Geranoaetus polyosoma*) inhabiting synanthropic and wildlife environments from Brazil and Chile, respectively. Whole-genome sequence (WGS) analysis revealed that *E. coli* UNB7 and GP188 belonged to the globally disseminated clone ST602, carrying a wide resistome against antibiotics (β-lactams), heavy metals (arsenic, copper, mercury), disinfectants (quaternary ammonium compounds), and pesticides (glyphosate). Additionally, *E. coli* UNB7 and GP188 strains harbored virulence genes encoding hemolysin E, type II and III secretion systems, increased serum survival, adhesins and siderophores. SNP-based phylogenomic analysis, using an international genome database, revealed genomic relatedness (19–363 SNP differences) of GP188 with livestock and poultry strains, and genomic relatedness (61–318 differences) of UNB7 with environmental, human and livestock strains (Table S1), whereas phylogeographical analysis confirmed successful expansion of ST602 as a global clone of One Health concern. In summary, our results support that ESBL-producing *E. coli* ST602 harboring a wide resistome and virulome have begun colonizing wild birds in South America, highlighting a potential new reservoir of critical priority pathogens.

## Highlights

• ESBL-producing bacteria in wild birds from South America have been investigated.
• Colonization of wild birds by CTX-M-producing *E. coli* is highlighted.
• Global *E. coli* ST602 clone carrying wide resistome and virulome was identified.
• Phylogenomics revealed relatedness with animal, environmental and human strains.
• Phylogeographical analysis confirmed ST602 as a global clone of One Health concern.

## Graphical abstract

**Figure:**
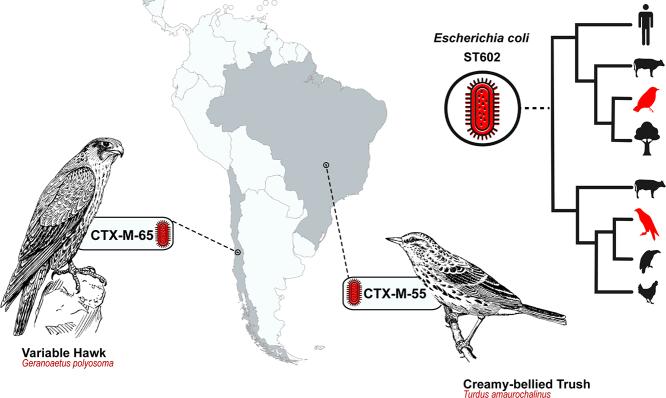

## 1 Introduction

Antibiotic resistance occurs naturally, however, the overuse and misuse of antibiotics in human and veterinary medicine, as well as in agriculture, livestock and animal husbandry have accelerated the process [1]. In addition to the selective pressure from antibiotics, resistance can also develop due to selective pressures from disinfectants (e.g. quaternary ammonium and triclosan), pesticides, and heavy metals, which are released into the environment by human activity [2].

Specifically, extended-spectrum β-lactamase (ESBL)-producing Enterobacterales have become an increasing public health issue worldwide, being recognized as critical priority pathogens by the World Health Organization [3]. Currently, ESBL-positive pathogens have been identified in companion and wild animals, becoming therefore a One Health problem [4,5]. ESBL enzymes confer resistance to both human and animal broad-spectrum cephalosporins. Among ESBLs, those of CTX-M family are the most widespread, and clinically relevant. Genes encoding ESBLs are often found on plasmids, which has enabled their spread, contributing to persistence and global dissemination of high-risk clones [6].

Noteworthy, wild animals have emerged as novel potential reservoirs and spreaders of antibiotic-resistant priority pathogens, since they have been directly exposed to polluted environments, and their feces are freely dispersed, possibly contaminating surface waters and soils [7]. While wildlife has been overlooked in the epidemiology of medically important antibiotic-resistant bacteria, isolation of ESBL-producing *Escherichia coli* from wild birds has begun to be reported worldwide, deserving epidemiological attention [7,8].

We hereby report microbiological and genomic characteristics of ESBL-producing *E. coli* colonizing wild birds in Brazil and Chile, highlighting its potential as spreaders of CTX-M genes in South America. In this regard, resistome (antibiotics, heavy metals, pesticides, and disinfectants), virulome and clonal relatedness have been investigated in depth.

## 2 Material and methods

### 2.1 Sample collection, bacterial identification and antimicrobial susceptibility testing

Between 2017 and 2019, a cross-sectional surveillance study was conducted to investigate the occurrence and genomic features of extended-spectrum β-lactamase (ESBL)-producing bacteria in wild birds in Chile and Brazil. A total of 118 wild birds belonging to Accipitriformes (*n* = 5), Anseriformes (*n* = 3), Apodiformes (*n* = 1), Charadriiformes (*n* = 6), Columbiformes (*n* = 5), Coraciiformes (*n* = 3), Cuculiformes (*n* = 1), Galbuliformes (*n* = 3), Nyctibiiformes (*n* = 1), Passeriformes (*n* = 78), Piciformes (*n* = 5), Psittaciformes (*n* = 2), Strigiformes (*n* = 1), Opisthocomiformes (*n* = 2), Tinamiformes (*n* = 1), and Trogoniformes (*n* = 1) orders, were captured by mist net. Cloacal samples were aseptically collected and maintained in Amies transport medium with charcoal (Absorve®), at room temperature, until processed. Swabs were inoculated onto MacConkey agar plates supplemented with ceftriaxone (2 μg/mL) and incubated overnight at 35 ± 2 °C for 18 h [9]. Colonies were individually selected for identification using Matrix Assisted Laser Desorption Ionization Time of Flight Mass Spectrometry (MALDI-TOF MS, Bruker Daltonik). Antimicrobial susceptibility testing was performed by the Kirby-Bauer disc diffusion method using the Clinical Laboratory Standards Institute guidelines [10], using amoxicillin/clavulanate (20/10 μg), ceftriaxone (30 μg), cefotaxime (30 μg), ceftiofur (30 μg), ceftazidime (30 μg), cefepime (30 μg), cefoxitin (30 μg), imipenem (10 μg), ertapenem (10 μg), meropenem (10 μg), nalidixic acid (30 μg), ciprofloxacin (30 μg), enrofloxacin (5 μg), gentamicin (10 μg), tobramycin (10 μg), amikacin (30 μg) and chloramphenicol (30 μg). Extended-spectrum beta-lactamase (ESBL) production was screened by using the double-disc synergy test (DDST) [11], using amoxicillin/clavulanate, ceftriaxone, ceftazidime, cefotaxime and cefepime.

### 2.2 Whole genome sequence analysis of ESBL-producing *E. coli*

The total genomic DNA of ESBL producers *E. coli* strains was extracted and used to construct a paired-end library, which was sequenced using the platform Illumina MiSeq with 2 × 300 bp sequence length (Illumina, San Diego, California, US). De novo genome assembly was carried out using Unicycle v.0.4.7, and annotated by the NCBI Prokaryotic Genome Annotation Pipeline (PGAP) (https://www.ncbi.nlm.nih.gov/genome/annotation_prok/). Multilocus sequence type (MLST), plasmid replicons, resistome and serotype were identified using MLST v2.0, PlasmidFinder v2.1, ResFinder v3.2, and SerotypeFinder v2.0 tools, respectively, from the Center for Genomic Epidemiology (http://www.genomicepidemiology.org/). The *E. coli* phylogroup was determined in silico using the Clermont typing tool (https://www.iame-research.center/). Clinically important virulence factors were predicted and compared by ABRicate v0.9.8 (https://github.com/tseemann/abricate) using data from the *E. coli* Virulence Factors (https://github.com/phac-nml/ecoli_vf) and the Virulence Factor Database (VFDB) (http://www.mgc.ac.cn/VFs/). Heavy metal (HM) and biocide genes were detected using the BacMet2 database (http://bacmet.biomedicine.gu.se). For detection of pesticide resistance genes, contigs were aligned against our in-house database containing genes conferring resistance to glyphosate (*phnABCDEFGHIJKLMNOP*), atrazine (*atzABCDEF*) or organophosphate (*mpd* and *opd*). Threshold ID and minimum length values (identity and coverage) of 90% were used for gene prediction.

### 2.3 Phylogenomic analysis

Genome assemblies of 266 *E. coli* strains belonging to ST602 and their metadata were retrieved from the *Escherichia*/*Shigella* Enterobase database (https://enterobase.warwick.ac.uk). ABRicate 1.0.1 (https://github.com/tseemann/abricate) was used with CGE Resfinder 4.1 database (https://bitbucket.org/genomicepidemiology/resfinder) for screening of antimicrobial resistance genes in the 266 publicly available retrieved genomes, the two genomes obtained in our study (UNB7 and GP188), and an additional genome obtained from an *E. coli* ST602 (Pk-12 strain) isolated from an Eurasian coot *Fulica atra*, in Pakistan. Identity and coverage threshold were set to 90% and 95%, respectively. CSI Phylogeny 1.4 (https://cge.cbs.dtu.dk/services/CSIPhylogeny) was used with default settings to build an approximately maximum-likelihood phylogenetic tree of UNB7, GP188 and Pk-12 strains, along to the 266 genome assemblies retrieved from Enterobase. The genome of *E. coli* HB-Coli0 strain (ST602) was used as reference (RefSeq assembly accession: GCF_002116715.2), and iTOL (https://itol.embl.de) was used to midpoint rooting the generated tree, to annotate the tree with Enterobase metadata, and to delete from the tree strains that lacked country and/or source of isolation in Enterobase metadata. iTOL was also used to build heatmaps indicating presence/absence of resistance genes for each antimicrobial class based on data generated by ABRicate and Resfinder 4.1 phenotype predictions, as well as presence/absence of resistance genes found by ABRicate in genomes of strains inside clades containing isolates from wild birds (including UNB7, GP188 and Pk-12 strains).

## 3 Results

Among 118 cloacal swabs obtained from wild birds, two ceftriaxone-resistant *E. coli* strains (UNB7 and GP188) were isolated from a Creamy-bellied Thrush (*Turdus amaurochalinus*) and a Variable hawk (*Geranoaetus polyosoma*) (Table 1). The Creamy-bellied Thrush was captured in a university campus in Brasilia, Midwest Brazil, whereas the Variable hawk was captured near the Andean mountain range in Chillán, Chile.

**Table 1:** Epidemiological data, resistome and plasmidome of CTX-M-55- and CTX-M-65-producing *Escherichia coli* belonging to the pandemic clone ST602 isolated from wild birds (*Turdus amaurochalinus* and *Geranoaetus polyosoma*) in Brazil and Chile.

Characteristics	*E. coli* strain UNB7	*E. coli* strain GP188
Host	*Turdus amaurochalinus*	*Geranoaetus polyosoma*
Country	Brazil	Chile
Epidemiological data		
Serotype	ONT:H21	ONT:H21
*fimH*	86	86
MLST (ST/CC)^a^	602/446	602/446
Phylogroup	B1	B1
Resistome		
Antibiotics		
β-lactams	*bla*_CTX-M-55_	*bla*_CTX-M-65_, *bla*_TEM-1B_
aminoglycosides	–	*aph(4)-Ia*, *aac(3)-IV*
quinolones	–	*gyrA* (D87N, S83L), *parC* (S80I)
tetracyclines	–	*tetB*
fosfomycin	–	*fosA7*
chloramphenicol	–	*florR*
Heavy metals		
arsenic	*arsBCR*, *glpF*	*arsBCR*, *glpF*
antimony	*arsBCR*, *glpF*	*arsBCR*, *glpF*
cadmium	*zinT*, *fieF*, *robA*, *zupT*, *ychH*, *ygiW*, *dsbAB*	*dsbABC*, *robA*, *ychH*, *ygiW*
cobalt	*rcnB*, *fecDE*, *fieF*, *zupT*	*fief*, *rcnABR*, *corAB*
copper	*bhsA*, *cutACEF*, *cueOR*, *cusCFRS*, *zupT*, *dsbC*	*cueOR*, *cutACEF*, *comR*, *bhsA*
chromium	*nfsA*	*nfsA*
iron	*fetAB*, *sitABCD*, *rcnA*, *rcnR*, *fieF*, *yqjH*	*yqjH*, *sitABCD*, *fetAB*
magnesium	*mntR*	*corAB*
mercury	*robA*	*robA*
zinc	*znuAB*, *zraR*, *zur*, *soxS*, *baeR*, *zntR*, *fieF*, *pitA*, *zupT*, *zitB*, *zinT*, *dsbAB*	*pitA*, *zitB*, *zupT*, *zinT*, *zntAR*, *znuAB*, *soxS*, *zur*, *zraR*
tellurite	*tehB*, *pitA*	*pitA*
tungsten	*baeR*	*baeR*
nickel	*nikABCDER*, *fieF*, *zupT*, *yqjH*	*nikABCDER*, *yqjH*, *corAB*
silver	*cusCFRS*, *robA*	*cusACFRS*
molybdopterin	*modBCE*	*modBCE*, *yieF*
Disinfectants		
QACs^b^	*acrEF*, *emrDK*, *mdtEFKN*, *tehA*	*emrDK*, *mdtBEFKNHPR*, *acrEF*, *tehAB*, *tolC*, *yji*, *cpxA*
hydrogen peroxide	*sitABCD*	*sitABCD*
Acidic or basic environment	*sodAB*	*gadCEWX*, *hdeAB*, *rpoS*, *ydeOP*, *yhcN*, *ymgB*, *yodD*, *sodAB*
Herbicides		
Glyphosate	*phnCDEFGHIJKLNOP*	*phnCDEFGHIJKLNOP*
Plasmids Inc-type (pMLST)^c^	IncF-type (F29:A-:B1), IncN, IncX	IncF-type (F18:A-:B1), IncI1-type (ST71/CC-7)
GenBank accession number	JAAVSK000000000	JAJNMH000000000

a MLST, multilocus sequence type; ST, sequence type; CC, clonal complex.

b QAC, quaternary ammonium compound.

c Plasmid multilocus sequence typing.

*E. coli* UNB7 exhibited resistance to ampicillin, cephalothin, cefotaxime, ceftiofur and ceftazidime, whereas *E. coli* GP188 displayed resistance to ampicillin, cephalothin, cefotaxime, ceftiofur, ceftazidime, nalidixic acid, ciprofloxacin, tobramycin, and chloramphenicol. Both isolates were ESBL producers, and belonged to sequence type ST602 (clonal complex CC446). Further genomic analysis of UNB7 revealed the presence of the *bla*_CTX-M-55_ ESBL gene and IncF-type, IncN, and IncX plasmids, whereas GP188 carried the *bla*_CTX-M-65_ gene and IncF-type and IncI1 plasmids. In addition to antibiotic resistance, *E. coli* strains displayed a wide virulome and resistome against disinfectants, heavy metals, and herbicides (Table 1 and Table 2).

**Table 2:** Virulome of CTX-M-55- and CTX-M-65-producing *Escherichia coli* belonging to the pandemic clone ST602 isolated from wild birds (*Turdus amaurochalinus* and *Geranoaetus polyosoma*) in Brazil and Chile.

Virulence factor		Virulome	
		*E. coli* strain UNB7 (Brazil)	*E. coli* strain GP188 (Chile)
Adhesion	Adhesins	*pixBCDFGHJ*, *ehaG*	*ehaAG*
	Fimbriae	*fimABCDEFGHI*, *stgABCD*, *ycbSTV*, *cfaA*, *matF*	*fimABCDEFGHI*, *cfaABCD*, *cah*, *matF*, *stgABCD*
	Pili	*pppA*, *cfaD*, *holfC*, *ppdABCD*, *ycbFR*, *ygdb*, *yggr*, *ecpABCD*	*ecpABCDR*, *hofBCQ*, *ppdABCD*, *yggr*, *ygdb*, *ycbFRSTUV*, *yagVWXYZ*
	Flagella	*flgABCDEFGHIJKLN*, *flhABCDE*, *fliAEFGHIJKLMNOPQRTYZ*, *motAB*, *flk*	*flgABCDEFGHIJKLN*, *flhABCDE*, *fliAEFGHIJKLMNOPQRSTYZ*, *motAB*, *flk*
	Curli fibers	*csgBCDEFG*	*csgBCDEFG*
	Type II secretion system	*gspCDEFGHIJKLM*, *yghG*	*gspCDEFGHIJKLM,yghG*
	Type III secretion system	*epaOPQRS*, *eprHIJK*, *espL, espR1*, *espX*	*epaOPQRS*, *eprHIJK*, *espL3*, *espL4*, *espR1*, *espX1*, *espX5*
	Type IV secretion system	*–*	*fha*, *hcp1*, *hcp2*, *clpV*
Toxins	Colicin	*cvaC*	*cvaC*
	Haemolysin	*hlyE*	*hlyE*
	Heat-stable enterotoxin	*–*	*astA*
	Chemotaxis	*cheABMRWYZ*	*cheABRWYZ*, *tar*
	Increased serum survival	*iss*	*iss2*
	Invasion of brain endothelium	*ibeA*	*ibeBC*
Siderophores	Metal ion binding	*sitABCD*	*sitABCD*
	Aerobactin	*iucABCD*, *iutA*	*iucABCD*, *iutA*
	Enterobactin	*entABCEFS*, *fes*	*entABCEFS*, *fes*
	Ferrienterobactin	*fes*, *fepABCD*	*fes*, *fepABCD*
	Iron-uptake	*iroBCDEN*	*iroBCDEN*, *ireA*

**Fig. 1:**
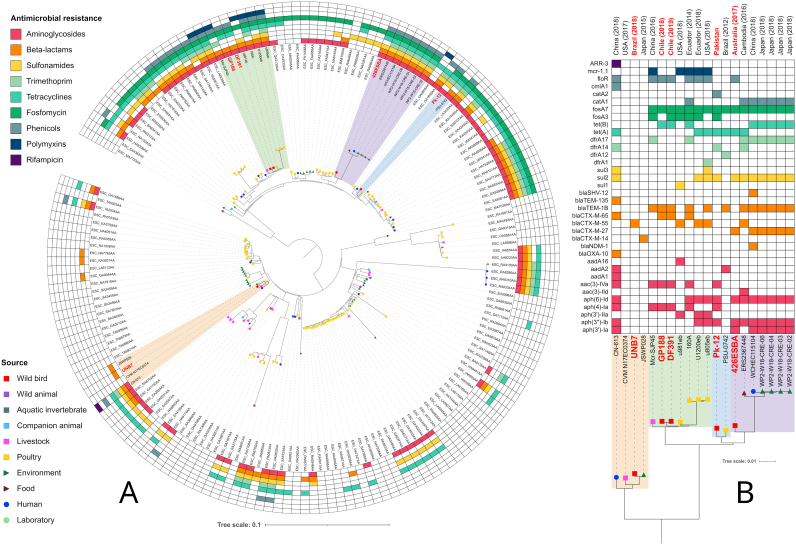
In A, phylogenomic tree of *Escherichia coli* ST602 strains, their source and presence/absence of drug resistance genes for 9 antimicrobial classes. Wild bird isolates are indicated in red, and their clades are highlighted. Strains in highlighted clades were labelled by their name, and the others were labelled by Entrerobase ID (Uberstrain). In B, resistomes of isolates in the clusters formed with each wild bird isolate. (For interpretation of the references to colour in this figure legend, the reader is referred to the web version of this article.)

Comparative phylogenetic analysis clustered UNB7 with human (318 SNPs difference), livestock (220 SNPs difference), and environmental (61 SNPs difference) *E. coli* strains of ST602, identified in China, USA, and Japan, respectively. Moreover, while GP188 strain was closest related (15 SNPs difference) to an *E. coli* strain isolated from a Chilean Andean condor, SNP differences with poultry strains from Ecuador and United States of America (USA) and a livestock strain from China, ranged from 106 to 385 SNPs (Fig. 1, Table S1)

## 4 Discussion

In this study, we identified two *E. coli* strains producing CTX-M-55 or CTX-M-65 ESBLs, in wild birds from South America. In this regard, CTX-M-55-positive *E. coli* have been mostly identified in human and animal hosts from Asian countries [12,13], and less frequently from European and North American countries [[14], [15], [16], [17], [18]]. In South America, CTX-M-55-producing *E. coli* have been identified in human host, poultry, peri-urban wild animals and water samples, in Ecuador and Brazil [[19], [20], [21], [22]]. CTX-M-65-producing *E. coli* have been mostly reported in human and animal hosts from Asian countries, mainly China and Korea [[23], [24], [25], [26]], whereas in Europe, North America, and Oceania there are fewer reports restricted to human hosts [[27], [28], [29]]. In South America, *E. coli* carrying CTX-M-65 ESBL genes have been identified in humans and wild bids in Bolivia and Chile, respectively, and in a giant anteater in a zoo, in Brazil [[30], [31], [32], [33]].

**Fig. 2:**
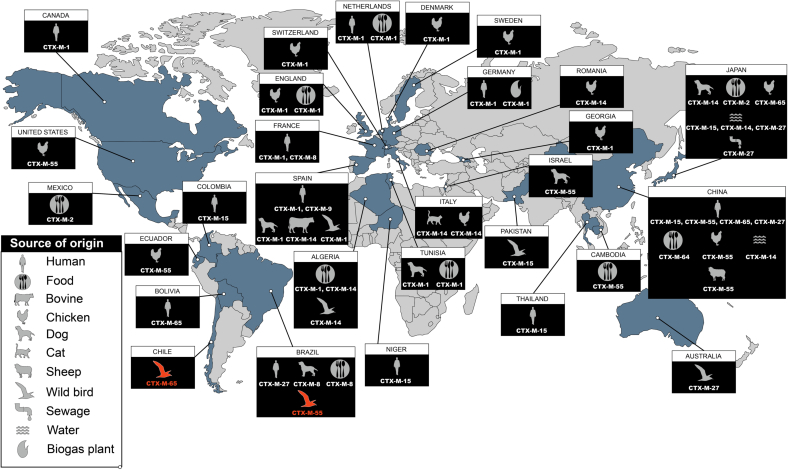
Worldwide distribution of CTX-M-producing *E. coli* belonging to ST602. To data, CTX-M-producing *E. coli* ST602 have been identified in Algeria, Australia, Bolivia, Brazil, Cambodia, Canada, Chile, China, Denmark, Ecuador, England, France, Germany, Georgia, Italy, Japan, Netherlands, Niger, Mexico, Pakistan, Romania, Spain, Sweden, Switzerland, Tunisia, Thailand and United States (Data were retrieved from Enterobase and from publicly available scientific literature, and quoted in the supplementary Table S2).

The MLST analysis showed that both CTX-M-55- and CTX-M-65-positive *E. coli* strains belonged to ST602 (CC446). This clone has been reported globally in humans, pets, wild and food-producing animals, and water and food samples. Specifically, CTX-M (−1, −2, −8, −9, −14, −15, −27, −55, −64, −65)-producing *E. coli* ST602 have been reported in Algeria, Australia, Bolivia, Brazil, Cambodia, Canada, Chile, China, Colombia, Denmark, Ecuador, England, France, Germany, Georgia, Israel, Italy, Japan, Mexico, Netherlands, Niger, Pakistan, Romania, Spain, Sweden, Switzerland, Thailand, Tunisia, and USA (Fig. 2, Table S2).

Phylogenetic analysis and comparative resistome from wild birds suggest that *E. coli* ST602 producing carrying CTX-M enzymes have been circulating in wild birds in Brazil, Chile, Australia, and Pakistan at least since 2017 (Fig. 1B). On the other hand, genomic relatedness between the CTX-M-65-positive *E. coli* strain GP188 and another CTX-M-65-positive *E. coli* strain (DF391), previously identified in Chile, was confirmed. Moreover, both isolates were clustered with four poultry *E. coli* isolates from Ecuador and USA, and a livestock isolate from China.

The CTX-M-55-producing *E. coli* strain UBN7 was closely related to an environmental isolate from Japan, being further clustered with a livestock strain from USA, and a human isolate from China. Although, the CTX-M-27-positive *E. coli* ST602, isolated from a Seagull in Australia, was not isolated in this study, our phylogenetic analysis also highlights the genomic relatedness with environmental, human, and food CTX-M-27-producing *E. coli* strains identified in Japan, China, and Cambodia, respectively.

Noteworthy, both strains identified in our study displayed a wide resistome against disinfectants, heavy metals, and herbicides. In this respect, the operon *phn*, which confers resistance to glyphosate (an herbicide largely used in agriculture, silviculture, and urban gardens) was identified. Although some bacteria, such as *Achromobacter* spp., *Ochrobactrum anthropi*, *Sinorhizobium meliloti*, *Rhizobium radiobacter*, and *Burkholderia pseudomallei*, utilize glyphosate as a source of phosphorus, *E. coli* is unable to use this herbicide as an inorganic phosphate (P_i_) source [34]. Therefore, the presence of the operon *phn* in UNB7 and GP188 could suggest an adaptative tolerance mechanism to pesticides. In fact, the wide resistome could be related to environmental pollution by anthropogenic activities, since agricultural and industrial activities, including fertilizer application and mining, have contributed to heavy metal and herbicide accumulation in the environment [35].

*E. coli* strains UBN7 and GP188 also carried genes conferring tolerance to arsenic, antimony, cadmium, cobalt, copper, chromium, iron, magnesium, mercury, zinc, tellurite, tungsten, nickel, silver, and molybdopterin. Metal pollutants can be released into the environment from many sources, such as agriculture, battery recycling, and metal production processes, as they resist to degradation can persist in water and soil [36]. Specifically, heavy metal contamination has been related with co-selection of other antimicrobial resistance genes and potentially contributes to the spread of antibiotic resistance [[37], [38], [39]]. Additionally, both isolates carried genes conferring tolerance to quaternary ammonium compounds (i.e. *emrDK*, *mdtEFKN, acrEF*, *tehAB*). Therefore, the extensive use of disinfectants in industry, hospitals, domestic households, and cosmetic products may be imposing a selective pressure [40].

Virulome analysis confirmed the presence of genes related to adherence (*pix* and *eha*), increase serum survival (*iss*), invasion (*ibeA*), iron uptake (*iroBCDEN*), chemotaxis (*cheABMRWYZ*), homeostasis (*cadA*), type II secretion system (*gspCDEFGHIJKLM* and *yghG*), type III secretion system (*epaOPQRS, eprHIJK, espL, espR1, espX*), curli fibers (*csgBCDEFG*), metal ion binding (*sitABCD*), colicin (*cva*), aerobactin (*iucABCD* and *iutA*), enterobactin (*entABCEFS*), ferrienterobactin (*fes* and *fepABCD*), and hemolysin E (*hlyE*). Thus, we contribute to knowledge virulence-related genes that are circulating in *E. coli* colonizing wild birds.

As a limitation of this study, a relatively small number of wild birds were sampled, and it was not possible to determine exactly how these animals acquired ESBL-producing *E. coli*. Unfortunately, there is a lack of information regarding the environmental factors and mechanisms that facilitate the transmission of *E. coli* strains from wildlife environments to synanthropic environments. However, it is well-known that *E. coli* is normally found in the intestinal tract of vertebrates, being widely used as an indicator of faecal contamination of food and water [41]. Therefore, transmission of antimicrobial-resistant *E. coli* from anthropogenically polluted environments to wildlife environments can occur dynamically and continuously. Although, antibiotic-resistant *E. coli* have been reported in wild birds at least since 1978 [42], it is not clear how ESBL-producing *E. coli* make their way into the wildlife environment. Most likely, ESBL-positive *E. coli* can reach the environment from hospital and/or community pollution [41,[43], [44], [45], [46]], whereas acquisition of antimicrobial-resistant bacteria by wildlife is probably mediated by horizontal gene transfer on conjugative plasmids, from clinical isolates, or from the intake of resistant bacteria from aquatic environments polluted by industrial, agricultural and domestic waste [44,45,47].

## 5 Conclusions

In conclusion, we report the identification and genomic features of two ESBL (CTX-M-55 and 65)-producing *E. coli* colonizing wild birds in countries with endemic occurrence of human infections caused by CTX-M producers, highlighting new potential reservoirs of critical priority pathogens. *E. coli* strains belonged to ST602, a lineage of global distribution. Worryingly, our epidemiological tracking revealed global dissemination of this clone at the human-animal-environment interface. Additionally, we report that ST602 isolated from wild bird species has been harboring CTX-M enzymes at least since 2017. Specifically, the wide resistome of CTX-M-55 and CTX-M-65-positive *E. coli* strains ST602, for clinically relevant cephalosporins, disinfectants, heavy metals, and herbicides, could denote environmental pollution by anthropogenic activities related to the use of these antimicrobial and biocides compounds. Therefore, these data provide important information to be used in epidemiological studies of critical ESBL-producing pathogens within a One Health perspective, as well as to understand genomic aspects related to adaptation and dissemination of critical priority pathogens at the human–animal-wildlife interface. Hence, we strongly encourage continuous surveillance of ESBL-producing *E. coli* in wild birds in Latin America for a better comprehension of the transmission pathways and clinical impacts of such pathogens in wildlife populations.

## Accession numbers

The datasets presented in this study can be found in online repositories. Both GP188 and UNB7 Genome shotgun projects have been deposited at DDBJ/ENA/GenBank under the accession JAJNMH000000000 and JAAVSK000000000, respectively. Additionally, genomic and epidemiological information of both *E. coli* strains have been deposited at OneBR (EcBr) platform (http://onehealthbr.com), under IDs ONE133 (GP188) and ONE10 (UNB7), respectively.

## Author contributions

GD, DF, EM and NL designed the experiments. DF, BM, LS, and LP performed the sampling campaign. GD, DF, BC, FE and QM performed the experiments. GD, HF, EP and DF performed the WGS and Phylogenetic analyses and images. GD prepared the manuscript. All authors discussed the results, reviewed and edited the manuscript, and read and approved the final version of the manuscript.

## Funding

This study was supported by the 10.13039/100000865Bill and Melinda Gates Foundation (Grand Challenges Explorations Brazil OPP1193112). Under the grant conditions of the Foundation, a CC BY or equivalent license is applied to the author accepted manuscript version arising from this submission. Additionally, this study was supported by the 10.13039/501100001807Fundação de Amparo à Pesquisa do Estado de São Paulo (2020/08224-9 and 2019/15578-4), 10.13039/501100003593Conselho Nacional de Desenvolvimento Científico e Tecnológico (AMR Aperfeiçoamento de Pessoal de Nível Superior (88882.333054/2019-01). GD, HF and BC are a research fellow of 10.13039/501100002322CAPES (88882.333521/2019-01, 88887.506496/2020-00 and 88882.333054/2019-01, respectively). DF-C was a research fellow of 10.13039/501100002848Comisión Nacional de Investigación Científica y Tecnológica, (CONICYT BCH 72170436). FE is a research fellow of 10.13039/501100001807FAPESP (2019/15578-4). NL is a research fellow of 10.13039/501100003593CNPq (314336/2021-4).

## Ethical approval

Ethical approval was received from School of Veterinary Medicine and Animal Science of University of São Paulo (São Paulo, SP, Brazil) [no. 5625041119]. This study was carried out in compliance with the System Authorization and Information on Biodiversity (SISBIO) of the Brazilian Institute of Environment and Renewable Natural Resources [IBAMA; license number: 10013–5 and 57,944–4].

## Author statement

We declare that the manuscript ***“**CTX-M-producing Escherichia coli ST602 carrying a wide resistome in South American wild birds: another pandemic clone of One Health concern”* by Gislaine Dalazen, Danny Fuentes-Castillo, Luiz G. Pedroso, Herrison Fontana, Elder Sano Pereira, Brenda Cardoso, Fernanda Esposito, Quezia Moura, Bianca Santos Matinata, Luiz Fábio Silveira, Mashkoor Mohsin, Eliana Reiko Matushima, Nilton Lincopan has not been published before and is not under consideration for publication elsewhere.

All authors made relevant contributions to the development of the research, the manuscript has been read and approved by all named authors and confirm that the order of authors listed in the manuscript has been approved by all of us. We also affirm that there are no known conflicts of interest associated with this publication and there has been no significant financial support for this work that could have influenced its outcome.

We understand that the Corresponding Author is the sole contact for the Editorial process. He is responsible for communicating with the other authors about progress, submissions of revisions and final approval of proofs. We confirm that we have provided a current, correct email address.

## Declaration of Competing Interest

The authors declare that the research was conducted in the absence of any commercial or financial relationships that could be construed as a potential conflict of interest.

## Data Availability

Data will be made available on request.

## Appendix A Supplementary data

Table S1. SNPs matrix for *E. coli* strains belonging to ST602 Image 1

Table S2. Map data references reporting global distribution of E. coli ST602 Image 2
